# Towards Profiles of Resistance Development and Toxicity for the Small Cationic Hexapeptide RWRWRW-NH_2_

**DOI:** 10.3389/fcell.2016.00086

**Published:** 2016-08-26

**Authors:** Michaela Wenzel, Pascal Prochnow, Catherine Mowbray, Cuong Vuong, Stefan Höxtermann, Jennifer J. Stepanek, H. Bauke Albada, Judith Hall, Nils Metzler-Nolte, Julia E. Bandow

**Affiliations:** ^1^Applied Microbiology, Ruhr University BochumBochum, Germany; ^2^Institute for Cell and Molecular Biosciences, Newcastle UniversityNewcastle upon Tyne, UK; ^3^AiCuris Anti-infective Cures GmbHWuppertal, Germany; ^4^Clinic for Dermatology and Allergology, St. Josef HospitalBochum, Germany; ^5^Chair of Inorganic Chemistry I - Bioinorganic Chemistry, Ruhr University BochumBochum, Germany

**Keywords:** antimicrobial peptide, toxicity tests, acute, antimicrobial cationic peptides, antimicrobial peptide resistance, toxicity mechanisms

## Abstract

RWRWRW-NH_2_ (MP196) is an amphipathic hexapeptide that targets the bacterial cytoplasmic membrane and inhibits cellular respiration and cell wall synthesis. In previous studies it showed promising activity against Gram-positive bacteria and no significant cytotoxicity or hemolysis. MP196 is therefore used as lead structure for developing more potent antibiotic derivatives. Here we present a more comprehensive study on the parent peptide MP196 with regard to clinically relevant parameters. We found that MP196 acts rapidly bactericidal killing 97% of initial CFU within 10 min at two times MIC. We were unable to detect resistance in standard 24 and 48 h resistance frequency assays. However, MP196 was effective against some but not all MRSA and VISA strains. Serum binding of MP196 was intermediate and we confirmed its low toxicity against mammalian cell lines. MP196 did neither induce NFκB activation nor cause an increase in IL8 levels at 250 μg/mL, and no IgE-dependent activation of basophil granulocytes was detected at 500 μg/mL. Yet, MP196 demonstrated acute toxicity in mice upon injection into the blood stream. Phase contrast microscopy of mouse blood treated with MP196 revealed a shrinking of erythrocytes at 250 μg/mL and severe morphological changes and lysis of erythrocytes at 500 μg/mL. These data suggest that MP196 derivatization directed at further lowering hemolysis could be instrumental in overcoming acute toxicity. The assessment of hemolysis is a critical step in the evaluation of the clinical potential of promising antimicrobial peptides and should be accompanied by microscopy-based morphological analysis of blood cells.

## Introduction

Resistance of microorganisms against antibiotics has developed into a major health problem. According to a 2015 report, antimicrobial resistance accounts for 700,000 deaths worldwide each year and numbers are rising (O'Neill, 2015). Multi-resistant bacteria, so-called superbugs, constitute a particular problem. In the US alone, multi-resistant *Staphylococcus aureus* (MRSA) cause over 100,000 life-threatening infections per year (Klevens et al., 2007) and in some European countries MRSA prevalence in blood cultures exceeds 50% (ECDC/EMEA Joint Working Group, 2009). Resistances against last line of defense antibiotics are on the rise (Stefani et al., 2015), while at the same time, antibiotic approval rates declined drastically (Spellberg et al., 2015). Therefore, novel antibiotic compounds and alternative antimicrobial strategies are urgently needed. Several drugs and therapeutic antibodies are currently in early clinical development (Vuong et al., 2016). Additional approaches to keeping up with the rapid development and spread of bacterial resistance include optimizing hospital hygiene (Köck et al., 2014), systematic MRSA decolonization (Köck et al., 2014), MRSA-selective phage therapy (Matsuzaki et al., 2003), or maggot therapy to clean wounds from necrotic tissue (Bowling et al., 2007). Several natural products with known antibacterial properties are evaluated for topical use as wound dressings against MRSA, e.g., oak extract or honey (Blaser et al., 2007; Kwakman et al., 2010; Sherlock et al., 2010; Graham, 2014). Seeking alternative or adjunct therapy options, antimicrobial peptides are currently re-evaluated. This compound class occurs naturally in all domains of life and is part of the innate immune system (Brogden and Brogden, 2011). Over 2500 peptides are currently listed in the Antimicrobial Peptide Database (http://aps.unmc.edu/AP/main.php), not even covering a fraction of the variety of synthetic peptides that have been produced over the last years. In addition to being antibacterial, many of these peptides possess antiviral, antifungal, anti-parasite, or anticancer activity and/or can act as immune enhancers or modulators (Mansour et al., 2014). Importantly, many peptides are hardly affected by bacterial resistance (Yeaman and Yount, 2003). Thus, antimicrobial peptides constitute an enormous resource of potential drug candidates.

While naturally occurring peptides are sometimes difficult to isolate or synthesize, smaller chemically designed peptides are an attractive alternative (Haug et al., 2004). Many natural peptides share a structural motif characterized by arginine (R) and tryptophane (W) residues. In an attempt to find the minimal pharmacophore of such RW-rich peptides, the hexapeptide RWRWRW-NH_2_ (MP196) was found to be the shortest RW-based sequence that still possesses good antibacterial activity (Strøm et al., 2003). Since many cationic antimicrobial peptides are cytotoxic and/or hemolytic, clinical application of these highly effective antimicrobials is limited (Jamasbi et al., 2014). MP196 was not significantly hemolytic or cytotoxic, and MP196—lipid interactions were shown to be highly selective for bacterial over mammalian membrane mimics (Albada et al., 2012a; Wenzel et al., 2014). Therefore, it was chosen as a peptide lead structure and subjected to several chemical modification approaches including organometallic (Albada et al., 2012a) and fatty acyl derivatization (Albada et al., 2012b) and multivalency studies (Hoffknecht et al., 2015, 2016). Several of the resulting compounds displayed drastically improved activity against both Gram-positive and Gram-negative pathogens but also enhanced hemolytic potential (Albada et al., 2012a,b). Systematic exchange of L- to D-amino acid residues resulted in optimized non-hemolytic linear peptides with excellent anti-MRSA activity (Albada et al., 2013, 2014). Importantly, neither organometallic nor fatty acyl modifications significantly changed the antibacterial mechanism of action compared to the parent peptide MP196, which integrates into the cytoplasmic membrane and by deforming the lipid bilayer delocalizes essential proteins involved in respiration and cell wall biosynthesis as well as cell division (Wenzel et al., 2014, 2016). Since it targets such an essential structure as the bacterial membrane and affects different membrane-associated processes at the same time, fast resistance development was not expected, making MP196-derived peptides interesting candidates for further antibiotic development.

In order to evaluate the potential of these short RW-rich peptides for antibacterial treatment and to chart new avenues for further structural optimization, we investigated resistance development and the toxicity profile of the original MP196 peptide as a representative of the whole group.

## Methods

### Antibiotics

MP196, FcPNA, and aurein 2.2 were synthesized as described previously (Chantson et al., 2006; Pan et al., 2007; Patra et al., 2010). Trimethoprim, rifampicin, and erythromycin were purchased from Sigma-Aldrich. MP196, FcPNA, rifampicin, and trimethoprim were dissolved in sterile DMSO, aurein 2.2 in sterile water, and erythromycin in ethanol. Unless otherwise noted, experiments were performed in biological duplicates.

### Minimal inhibitory concentration (MIC)

MICs were tested against *S. aureus* DSM 20231 (type strain), ATCC 43300 (MRSA), COL (MRSA), SG511 (VISA), and Mu50 (VISA) in Mueller Hinton (MH) broth in a microtiter plate assay according to Clinical and Laboratory Standards Institute (CLSI) guidelines. In short, serial peptide dilutions were prepared in MH, inoculated with 5 × 10^5^ colony-forming units (CFU) per mL, and incubated for 16 h at 37°C. Inoculated medium without peptide served as positive control, uninoculated medium as negative control. The lowest concentration that prevented visible growth was reported as MIC.

### Binding to bovine serum protein

Antibiotics were dissolved in 0.9% NaCl or undiluted bovine serum, respectively. MICs against *B. subtilis* 168 were determined on MH agar plates in a hole diffusion assay as described below. Plasma binding was determined by comparing MICs of the peptide incubated with and without bovine serum.

### Survival rates

MICs against *Bacillus subtilis* 168 were determined in a standard test tube assay as described previously (Wenzel et al., 2011). Survival rates were determined in Luria Bertani (LB) broth supplemented with 18 μg/mL MP196, 4 μg/mL FcPNA, 20 μg/mL aurein 2.2, or 0.18 μg/mL trimethoprim, respectively. Antibiotic solutions were inoculated with 5 × 10^5^ CFU/mL and aerobically incubated for 10 min at 37°C. Aliquots were plated on LB agar plates without antibiotic and incubated at 37°C for 16 h. Experiments were performed in technical duplicates and biological triplicates.

### Resistance frequency

MICs against *B. subtilis* 168 (*trpC2*) (Anagnostopoulos and Spizizen, 1960) were determined on agar plates in a hole diffusion assay. 10^8^ CFU were plated on MH agar plates and 100 μL of antibiotic dilutions (in 0.9% NaCl) were added to 6 mm holes. NaCl solution alone served as negative control. Plates were incubated at 37°C for 16 h. Notably, the MIC on MH agar plates (45 μg/mL) was much higher as compared to liquid MH (2 μg/mL). For determining resistance frequency, 10^8^ cells of *B. subtilis* 168 were plated on MH agar plates with four-fold MIC (180 μg/mL MP196, 3.2 μg/mL rifampicin, 2.4 μg/mL erythromycin) or plates without antibiotic. Plates were incubated at 37°C and evaluated after 24 and 48 h.

### Toxicity against mammalian cell lines

NRK-52E rat kidney epithelial cells were cultured in DMEM PAN (PAN Biotech, Cat.-Nr. P04-03500) and CCRF-CEM human T-cell lymphoblast cells in RPMI 1640 medium. Both media were supplemented with 10% calf serum (FCS), 100 U/mL penicillin, 100 μg/mL streptomycin, and 1% L-glutamine at 37°C in a 5% CO_2_ atmosphere. 2.5 × 10^3^ NRK-52E cells or 6.2 × 10^4^ CCRF-CEM cells were incubated with 200, 66, 22.2, 7.4, 2.4, 0.82, 0.27, and 0.09 μg/mL MP196 at 37°C for 72 h in a 5% CO_2_ atmosphere. Ten microliters of Alamar Blue (Invitrogen) were added to the plates and fluorescence was detected after 3 h with a Tecan Spectra Fluor Plus instrument (excitation at 550 nm, emission at 595 nm) as readout for cell viability. Each MP196 concentration was tested in technical and biological duplicates.

### Murine model of acute toxicity

Acute toxicity in mice was performed in accordance with the institutional proposal (M56/2012-A-02) approved by the Landesamt für Natur, Umwelt und Verbraucherschutz Nordrhein-Westfalen. Animal care and use was performed under federal guidelines. Mice were fed and received sterile water *ad libitum*. Animals were euthanized according to the institutional approval and federal guidelines. MP196 was solubilized in sterile 0.9% NaCl for intravenous injection into mice. A 5 mL/kg body weight volume was used to administer 5, 10, and 25 mg/kg body weight of the test compound or vehicle alone (0.9% NaCl) into the tail vein. In accordance with the animal welfare guidelines the N for these early toxicity tests was 1 animal per group. Mice were monitored for behavior and health conditions constantly for the first 2 h after each single injection. Monitoring was continued hourly between 3 and 8 h, at 16 h, and daily for 5 days after initial injection.

### NF-κB activation

RT4 bladder epithelial cells (ATCC HTB-2) were stably transfected with a plasmid construct containing an NF-κB promoter with a luciferase reporter. Cells were cultured in RPMI 1640 medium containing 10% FCS, 2.5% HEPES, 1% L-glutamine, and the eukaryotic antibiotic G418 (0.5 mg/mL, Sigma Aldrich) at 37°C in 5% CO_2_. Cells were passaged and seeded into 96-well white plastic clear-bottomed plates (Corning) at 5 × 10^4^ cells per well and allowed to grow for 48–72 h until confluent. Cells were then challenged with either flagellin (isolated from *E. coli*) at concentrations from 10 ng to 200 ng/mL as a positive control or the peptide MP196 at concentrations from 10 ng to 25 ug/mL, 250 and 500 μg/mL. Negative controls containing media only, 0.05% DMSO, or 0.25% DMSO were included. After challenge for 1, 6, and 24 h, cells were lysed using RLB (Promega) and stored overnight at −80°C before addition of luciferin (Promega). The resulting luminescence was read using an Omega Fluostar plate reader (BMG Labtech).

### IL8 induction

IL8 levels in RT4 bladder cells were measured with an ELISA assay. To this end, ATCC HTB-2 cells were cultured as described above and seeded into 12-well plastic plates at 2 × 10^5^ cells per well. Cells were grown for 72 h until confluent and challenged for 6 and 24 h with either 50 ng/mL flagellin (isolated from *E. coli*) as a positive control, with 0.25% DMSO as negative control, or with 250 μg/mL MP196. Media was collected post-challenge and IL-8 production was measured by ELISA (R&D Systems) as per manufacturer's instructions with concentrations as follows: capture antibody 4 μg/mL, detection antibody 20 ng/mL, media diluted 1 in 50 in 1x PBS with 0.1% BSA. A negative control and standard curve were included on each plate. Plates were read using an Omega Fluostar Plate reader (BMG Labtech) at 450 and 540 nm and concentrations calculated using blank-corrected data fitted to the standard curve using 4-parameter fit, after which the dilution factor was applied.

### Flow cytometric detection of basophil activation

Basophil activation was tested using the Basotest reagent kit (Glycotope Biotechnology) according to the manufacturer's instructions. This test involves a phycoerythrin (PE)-conjugated anti-IgE and a fluorescein isothiocyanate (FITC)-conjugated anti-CD63 monoclonal antibody. Heparinized whole blood from one donor was incubated with 1, 5, 10, 25, 100, or 500 μg/mL MP196 solution, 5% DMSO, or 2 μM N-formyl-Met-Leu-Phe (positive control). Anti-IgE-PE/anti-gp53-FITC (CD63)-stained cells were excited at 488 nm and fluorescence was measured with a FACSCanto II-cytometer equipped with FACSDiva software (Becton-Dickinson). About 1000 basophilic granulocytes expressing high amounts of IgE per sample were acquired by fluorescence triggering in the FL2 channel (PE). An analysis gate was set in the SSC/FL2 dot plot around basophilic cells. Markers were set for FL2 and FL1 was analyzed using the same marker positions. The percentage of cells expressing the antigen gp53 was analyzed in FL1 (FITC).

### Phase contrast microscopy of mouse blood

Blood was collected from surplus animals after sacrificing. The procedure has been approved by the Body of Internal Animal Welfare FNWI, University of Amsterdam. After sacrificing, whole blood was drained from mice into anticoagulant tubes (26.358, Sarstedt) and stored at −20°C until further use. One hundred and forty-six microliters of blood were mixed with 12.5 μL antibiotic dilution in 0.9% NaCl to give concentrations approximately corresponding to the acute toxicity experiments (given that a standard mouse weighs 20 g and has an average blood volume of 1.46 ml), namely 250 μg/mL MP196. In addition 500 μg/mL MP196 were tested. 0.9% NaCl and 5% DMSO were used as negative controls. Samples were microscopically examined after 30 min incubation at room temperature using a Nikon Eclipse Ti equipped with a CFI Plan Apochromat DM 100x phase contrast oil objective, an Intensilight HG 130 W lamp, a C11440-22CU Hamamatsu ORCA camera, and NIS elements software, version 4.20.01.

### Hemolysis of murine red blood cells

Mouse blood was obtained as described above for the phase contrast microscopy. The red blood cells were prepared from these murine samples as described in Albada et al. for human erythrocytes. The hemolytic activity of MP196 was determined as described in the same reference (Albada et al., 2012a). MP196 was tested at 250 and 500 μg/ml, and 5% DMSO and 1% triton X-100 served as negative and positive controls, respectively.

## Results and discussion

We determined the minimal inhibitory concentrations (MICs) against our model organism *B. subtilis* and several Gram-positive and Gram-negative bacterial strains of clinical relevance (Table 1). MP196 displayed decent activity against *B. subtilis*, the *S. aureus* type strain DSM 20231, the MRSA type strain ATCC43300, as well as the vancomycin-intermediate (VISA) strain SG511, which carries a *graS* mutation that renders this strain sensitive to antimicrobial peptides (Sass and Bierbaum, 2009). MIC values against the clinical *S. aureus* isolates COL (MRSA) and Mu50 (VISA strain) were four to eight-fold higher compared to the *S. aureus* type strain (DSM 20231), suggesting that MP196 is not effective against all MRSA. Activity against Gram-negative *E. coli* and *A. baumannii* was comparatively weak and MP196 was not active against *P. aeruginosa* PA01, which displays an intrinsically high antibiotic resistance.

**Table 1:** **Minimal inhibitory concentrations of MP196 against different bacterial strains determined according to CLSI guidelines (μg/mL)**.

***B. subtilis***	***S. aureus***					***E. coli***		***A. baumannii***	***P. aeruginosa***
**168**	**DSM20231 (type strain)**	**SG511 (VISA)**	**ATCC43300 (MRSA)**	**COL (MRSA)**	**Mu50 (VISA)**	**DSM30083 (type strain)**	**W3110**	**DSM30007 (type strain)**	**PA01**
2	16	16	8	96	64	32	>64	32	>64

Despite good *in vitro* activity, the *in vivo* activity of antibiotics in general but antimicrobial peptides in particular might be compromised by high affinity to blood serum proteins (Rolinson and Sutherland, 1965). Antimicrobial activity of MP196 in the presence of bovine serum was 50%, thus ~50% of MP196 is bound to serum proteins. Therefore, we would not predict serum binding to be a limiting factor.

An important property of successful antibiotics is how fast they are able to kill bacteria. The more rapidly an antibiotic acts, the more restricted are the possibilities for bacteria to adapt to and survive antibiotic stress. Therefore, we determined short-time survival rates 10 min after antibiotic treatment. At two-fold MIC MP196 was rapidly bactericidal against *B. subtilis* killing 97% of an inoculum of 5 × 10^5^ CFU/mL. This 3% percent survival is comparable with that after exposure to two-fold MIC of the pore-forming antimicrobial peptide aurein 2.2 (0% survival) and the membrane-targeting peptide mimic FcPNA (5% survival). The bacteriostatic folate biosynthesis inhibitor trimethoprim was included as control (95% survival).

Since MP196 targets bacterial membrane lipids (Wenzel et al., 2014) and displays fast killing kinetics, it appears likely that bacteria cannot easily develop resistance against this peptide antibiotic. We determined the resistance frequency of *B. subtilis* exposed to four-fold MIC for 24 and 48 h. In fact, no resistant colonies could be found. Rifampicin and erythromycin, antibiotics with single protein targets, served as controls displaying resistance frequencies of 1.35 × 10^−7^ and 2.5 × 10^−8^, respectively. These data show that resistance development is unlikely to be a major roadblock for further development of MP196-based antibacterial peptides. Past experience has taught us that with antibiotic use eventually comes antibiotic resistance, even for antibiotics targeting the membrane and other complex targets such as polymyxins, daptomycin, or vancomycin (Tenover, 2006; Falagas et al., 2010; Vilhena and Bettencourt, 2012) and we have no indication that MP196-based peptides will be an exception.

MP196 was only slightly hemolytic even at very high peptide concentrations and not significantly cytotoxic against human liver carcinoma, colon adenocarcinoma, and breast cancer cell lines (Albada et al., 2012a). In preparation of a mouse model for acute toxicity, we additionally determined cytotoxicity for human T-cell lymphoblasts and rat kidney epithelial cells. Using an Alamar Blue assay, no cytotoxic effects were observed up to 200 μM (192 μg/mL). All these findings are well in line with the high specificity of MP196 for bacterial over mammalian membrane mimics (Wenzel et al., 2014). Encouraged by this, we tested initial acute toxicity in mice. To this end, MP196 was directly injected into the tail vein. At low concentrations (5 and 10 mg/kg body weight) the mouse showed signs of excitement and indisposition with recovery within 25 min after injection. At 25 mg/kg body weight paralysis of the hind legs occurred and the mouse died within few minutes. This quick reaction points to an acute toxicity effect rather than direct cytotoxicity.

**Figure 1:**
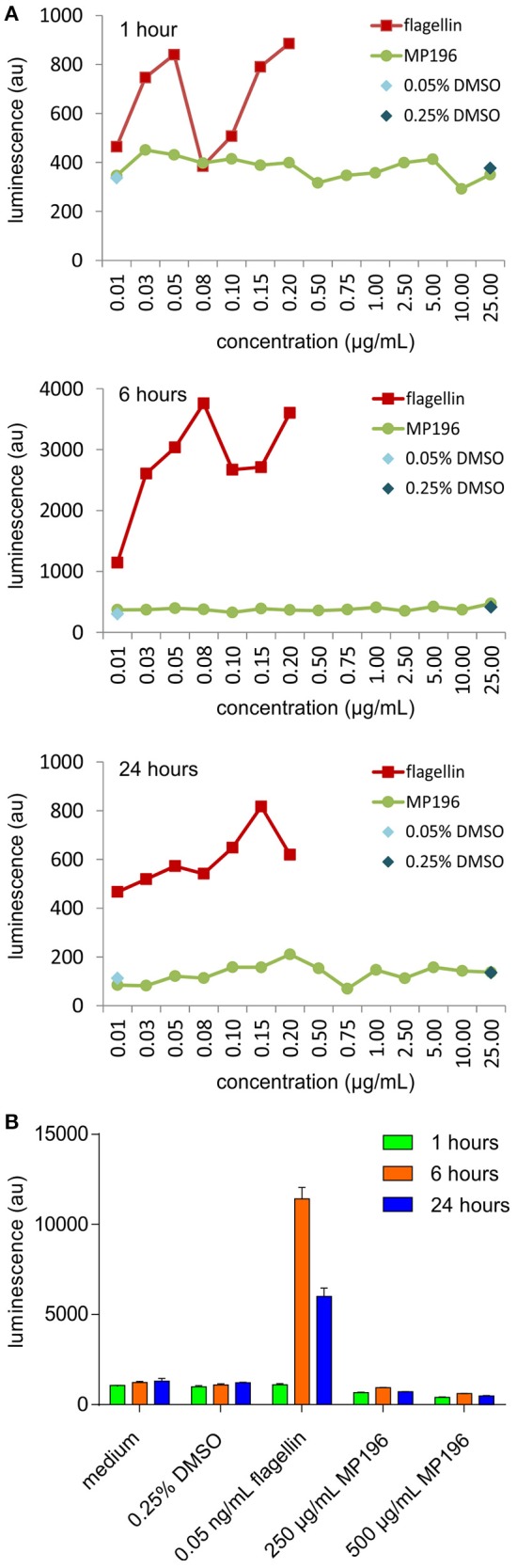
**Activation of Nf-κB in RT4 bladder cells at concentrations up to 25 μg/mL (A) and at 250 and 500 μg/mL (B)**.

**Figure 2:**
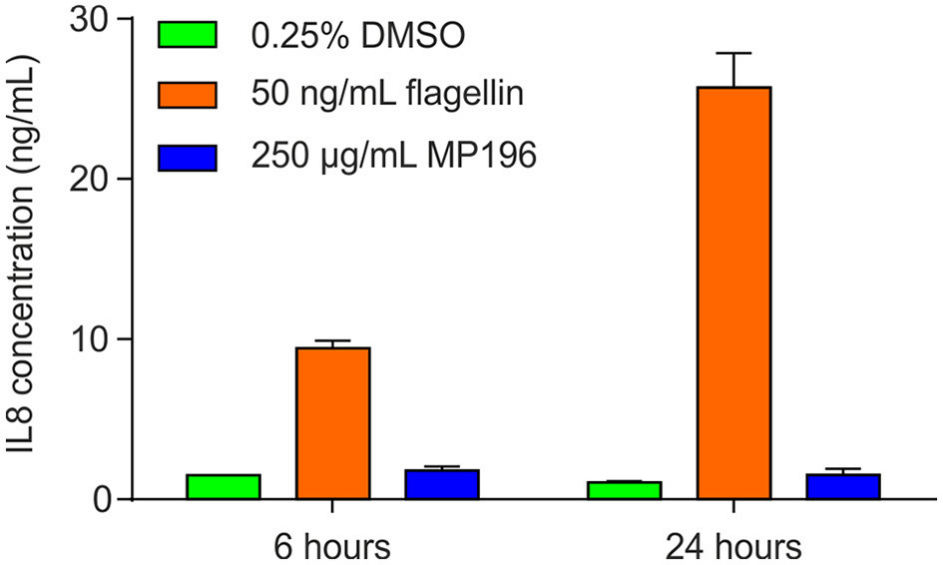
**IL8 levels in RT4 bladder cells challenged with MP196 and in DMSO and flagellin-treated controls**.

A potentially fatal immune reaction is the cytokine storm. Upon infection, cytokines are released by several immune cells to attract and activate further useful immune cells, such as T-cells or macrophages, which also secrete cytokines themselves (Janeway et al., 2001). While this feedback loop is normally carefully regulated, it can get out of control, especially when the body is facing a previously unknown immunogen, in the worst case leading to fatalities (Huang et al., 2005; Osterholm, 2005). Activation of NF-κB is a marker of cytokine release (Gilmore, 2006), but using RT4 reporter bladder cells, no NF-κB activation was observed at up to 25 μg/mL MP196 (12.5-fold MIC against *B. subtilis*; Figure 1A) and even at concentrations of 250 and 500 μg/mL no significant NF-κB activation was observed (Figure 1B). RT4 bladder cells further showed no elevated IL8 levels after challenge with 250 μg/mL MP196 (Figure 2). These data suggest that MP196 has no inflammatory activity when applied to epithelial cells.

**Figure 3:**
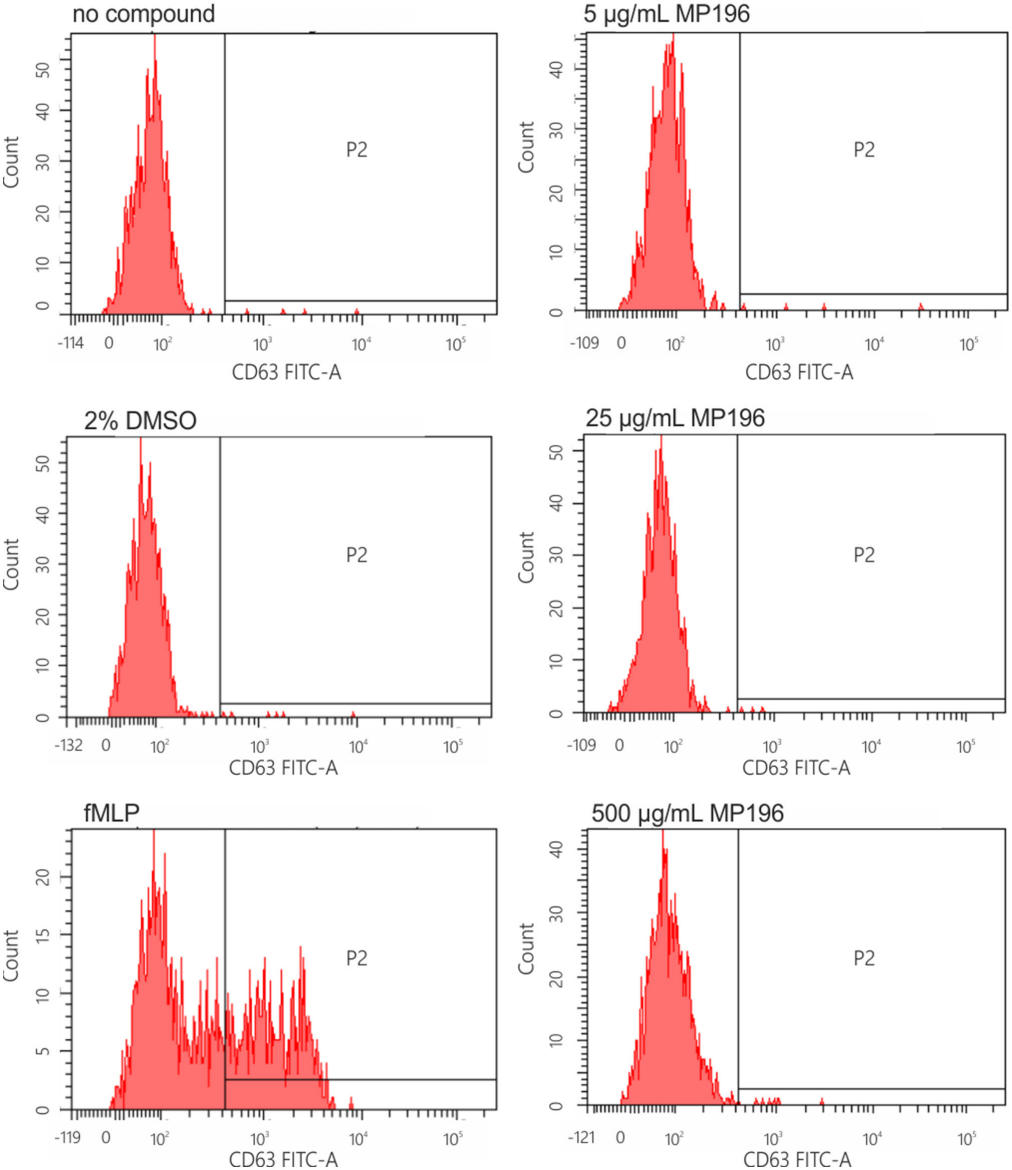
**IgE-dependent basophil activation measured by FACS**. Anti-IgE (PE) reacts with human IgE and is used for the characterization of basophilic granulocytes. Anti-gp53 (FITC) recognizes a glycoprotein expressed on activated basophils. The chemotactic peptide N-formyl-Met-Leu-Phe served as positive, DMSO as negative control.

Since the presence of external peptides might stimulate allergic reactions, we hypothesized that MP196-induced mortality might be caused by anaphylactic shock. Therefore, we tested the potential of MP196 to activate the release of histamine from immune cells. Histamine is stored in mast cells and basophil granulocytes and released upon cell activation by IgE (Janeway et al., 2001). IgE-dependent basophil activation was tested by flow cytometry using a two-color antibody reagent consisting of anti-IgE-PE and anti-gp53-FITC (CD63). This assay allows diagnosis of immediate-type hypersensitivity (type I reactions) and indicates histamine release. We performed this assay with blood from one donor and observed no IgE-dependent activation after addition of up to 500 μg/mL MP196 (Figure 3). While the preliminary tests are negative, it is important to note, that more extensive analyses would be needed to fully rule out allergic reactions.

**Figure 4:**
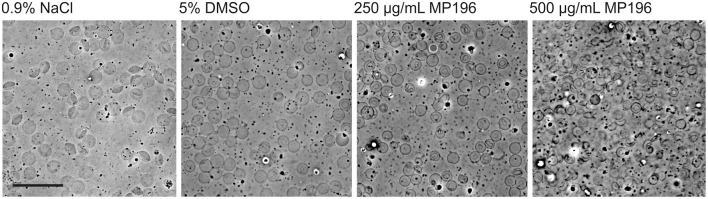
**Phase contrast images of whole mouse blood mixed with MP196**. Five percent DMSO is the maximum DMSO concentration reached by diluting MP196 from the stock solution. Scale bar represents 20 μm.

Since we had no evidence of MP196 triggering immune reactions, we attempted to determine solubility of MP196 in blood. Precipitated peptide could possibly clog blood vessels or promote agglutination of blood cells. While we could neither observe compound precipitation nor enhanced agglutination, we observed significant damage of blood cells (Figure 4). At 250 μg/mL, MP196 caused shrinking of erythrocytes. Further increasing the concentration to 500 μg/mL MP196 led to drastic morphological changes culminating in erythrocyte lysis. In a previous study, similar concentrations of peptide caused hemolysis of human erythrocytes of up to 17% at 500 μg/mL (Albada et al., 2012a). Using the same protocol described by Albada et al. we determined 14 and 23% lysis of murine erythrocytes at 250 and 500 μg/mL, respectively. Apparently, MP196 causes severe erythrocyte damage the severity of which was misjudged as “slight hemolysis” by a standard absorption-based hemolysis assay, suggesting that blood cells should also be monitored microscopically.

Taken together, two challenges for development of MP196 derivatives were identified in this study, one being the spectrum of activity and the second being toxicity toward blood cells. We believe that both challenges can be addressed by compound derivatization. First steps in this direction have been taken. The spectrum of activity has successfully been tackled by structural optimization programs. Adding a lipidated lysine side chain resulted in peptides with broad-spectrum and/or excellent anti-MRSA activity (Albada et al., 2012b). Lipidation also increased hemolysis, which has been addressed by systematically exchanging L- with D-amino acids resulting in non-hemolytic peptides with excellent activities against multi-resistant bacteria (Albada et al., 2013). The next step will be to investigate some of these advanced derivatives with regard to their toxicity.

## Conclusions

MP196 is a promising antibiotic lead structure for it kills bacteria rapidly, and, due to its multiple mechanisms, is not prone to be compromised by resistance development due to point mutations. All MP196 derivatives tested so far displayed the same mechanism of action as the parent compound (Wenzel et al., 2014, 2016), giving reason to believe that the concomitant advantages will be maintained in further derivatives. Serum binding is moderate and not perceived to become a limiting factor. MP196 is not equally effective against all MRSA or VISA strains and only weakly active against Gram-negative bacteria. Most importantly, acute toxicity was observed in mice dosed intravenously, likely as a result of toxicity toward erythrocytes. Structural optimization programs exploring peptide lipidation and L- to D-exchange show that peptides with broad-spectrum and/or excellent anti-MRSA activity (Albada et al., 2012b) and very low hemolytic activity (Albada et al., 2013) can be obtained. As the next step toward *in vivo* efficacy studies, these derivatives should be investigated for their effects on erythrocytes and, where appropriate, tested for acute toxicity.

## Author contributions

MW planned and performed experiments, analyzed data, and wrote the paper. CM, JH, CV, and SH planned and performed experiments and analyzed data. PP and JS performed experiments. HA and NM synthesized MP196 and provided input to the paper. JB planned and supervised the study and provided critical input on the manuscript.

### Conflict of interest statement

The authors declare that the research was conducted in the absence of any commercial or financial relationships that could be construed as a potential conflict of interest.
